# Stable Tetravalent Metal–Organic Frameworks for Electrocatalysis and Aqueous Electrochemical Energy Storage

**DOI:** 10.1021/acsami.5c20040

**Published:** 2025-12-26

**Authors:** Chou-Hung Hsueh, Chung-Wei Kung

**Affiliations:** † Department of Chemical Engineering, 34912 National Cheng Kung University, Tainan 70101, Taiwan; ‡ Program on Key Materials, Academy of Innovative Semiconductor and Sustainable Manufacturing, National Cheng Kung University, Tainan 70101, Taiwan

**Keywords:** Battery, Cerium-based MOF, Group 4 metal, Titanium-based MOF, Supercapacitor, Zirconium-based MOF

## Abstract

Owing to their chemical stability in water, relatively high specific surface area, and high tunability in both pore structures and chemical functionality in their pores, metal–organic frameworks (MOFs) constructed from tetravalent metal-based nodes, including zirconium-based MOFs, titanium-based MOFs, hafnium-based MOFs, cerium­(IV)-based MOFs and thorium-based MOFs, have been widely explored for various applications requiring humid or aqueous environments. In particular, over the past ten years, these MOFs have been widely employed in various aqueous electrochemical applications, including water splitting, oxygen reduction, carbon dioxide reduction, ammonia production, electrocatalytic sensing, aqueous supercapacitors, zinc-ion batteries, and vanadium flow batteries. Classified by the type of applications and the role that MOFs could play, progress in this research field is overviewed. Limitations and opportunities in this field for future studies are discussed.

## Graphical abstract

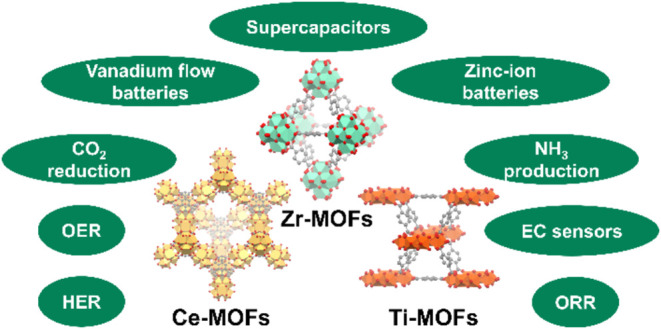

## 1 Introduction

As nanoporous materials emerging over the past three decades, metal–organic frameworks (MOFs) with diverse structures at the molecular scale have been extensively explored for a range of applications.^1^ The ultrahigh specific surface area and interconnected porosity of MOFs enable the immobilization of spatially accessible active sites with a high density, which renders MOFs attractive materials for heterogeneous catalysis.^2^ Thin films of MOFs deposited on electrodes are thus considered as ideal candidates for electrocatalysis, electrochemical energy storage, and electrochemical sensors.^3–9^ Electrochemically active sites or functional groups incorporated in the porous MOF thin film can be fully accessible to targeted ionic species/reactants coming from the external electrolyte, once the pore size of MOF is sufficiently larger than the size of targeted ions.

One major concern of employing MOFs in electrochemical systems is their poor chemical stability in water.^10^ Most MOFs may undergo structural degradation in aqueous solutions or even humid environments.^11,12^ It could lead to the dissolution of framework into ionic species, or the transformation of MOF into MOF-derived materials that do not possess the porosity with long-range order anymore.^13,14^ Such degradations of MOFs are sometimes more significant in the presence of applied potential, especially for those MOFs constructed from redox-active metal-based nodes.^15,16^ However, several electrochemical systems require the use of aqueous electrolytes with certain applied potentials. For example, most electrocatalysts need to initiate reactions in aqueous solutions, aqueous samples are the most common targets for electrochemical sensors, and energy-storage devices such as zinc-ion batteries and some supercapacitors employ aqueous electrolytes. Aqueous solutions are not used in lithium, sodium, and potassium-based batteries, but these devices require operations within a wide potential window. The structural stability of MOFs thus strongly hinders their use in electrocatalysis, electrochemical sensors, and energy storage.^15,16^ Compared to using pristine MOFs as active materials, serving MOFs as *in situ* consumed precursors or precatalysts,^16^ or directly employing MOF-derived materials,^17,18^ has been more frequently explored for electrochemical applications in literature.

Owing to their strong metal-to-ligand bonds, utilizing high-valent metal ions and carboxylate-based linkers is a common strategy to design and synthesize highly stable MOFs.^11,12,19^ Tetravalent MOFs constructed from Ti­(IV), Zr­(IV), Hf­(IV), Ce­(IV) or Th­(IV)-based nodes thus become appealing candidates for electrochemical applications while preserving their structural integrity.^19^ The earliest reported example, also one of the most extensively explored MOFs among them, is Zr­(IV)-based UiO-66, discovered by Lillerud et al. in 2008.^20^ Lots of structurally diverse Zr­(IV)-based MOFs (Zr-MOFs) with various linkers, topologies and pore sizes have thereafter been reported and investigated since then.^21,22^ Although their chemical stability depends on their structures and node connectivity, in general, these Zr-MOFs are highly stable in aqueous solutions from strongly acidic to weakly alkaline conditions.^12,23^ On the other hand, the first Ti­(IV)-carboxylate-based MOF, MIL-125­(Ti), was first reported by Férey et al. in 2009.^24^ Although there are not that many structurally diverse Ti­(IV)-based MOFs (Ti-MOFs) in literature compared to Zr-MOFs, owing to the difference in electronic configuration of Ti^4+^ compared to Zr^4+^, Ti-MOFs possess unique redox activity between Ti^4+^ and Ti^3+^ occurring on a part of their titanium atoms.^25^ It thus renders Ti-MOFs especially attractive for photocatalysis and photoelectrochemical systems.^26,27^ It is worth mentioning that the exchange of zirconium ions in the cluster of Zr-MOFs by titanium ions was sometimes reported, but in most cases, it should be realized as the postsynthetic grafting of titanium ions into Zr-MOFs.^28,29^ Hafnium atoms have very similar electronic configurations and ionic radius compared to zirconium atoms. Thus, several Hf­(IV)-based MOFs (Hf-MOFs) which are isostructural to their zirconium-based analogs have been reported.^30^ Since the dissociation enthalpy of the hafnium–oxygen bond (802 kJ mol^–1^) is higher than that of the zirconium–oxygen bond (776 kJ mol^–1^),^30^ Hf-MOFs are usually considered as slightly more stable options than Zr-MOFs. On the other hand, several Ce­(IV)-based MOFs (Ce-MOFs) that are isostructural to their zirconium-based analogs have been studied,^30,31^ and these materials possess similar chemical stability compared to Zr-MOFs.^32^ One noticeable feature of Ce-MOFs is the redox activity between Ce^4+^ and Ce^3+^ in a minor proportion of cerium atoms in their clusters, rendering them redox-active and electrochemically active.^33–35^ Thorium (Th­(IV))-based MOFs (Th-MOFs) belong to another emerging subclass of tetravalent MOFs with better chemical stability in alkaline solutions, but slightly worse stability in acidic conditions compared to their zirconium-based analogs.^32,36^

More specifically, the Pearson’s hard/soft acid/base (HSAB) principle provides a mechanistic explanation for these differences in the stability of MOFs.^37^ As hard Lewis acids, Zr­(IV) and Hf­(IV) form strong M–O­(carboxylate) bonds, giving rise to the highest hydrolytic stability among tetravalent MOFs.^38^ Ce­(IV)-based MOFs exhibit comparable stability but also feature the node-centered Ce^4+^/Ce^3+^ redox couple, as introduced previously.^35,39^ With a similar structural robustness, the redox activity of Ti­(IV) centers in Ti-MOFs has also been observed and utilized in photocatalytic and electrochemical systems.^26,27,40,41^ Finally, Th­(IV), with its large ionic radius and high coordination number, can generate highly open and interconnected frameworks.^42^ These intrinsic metal-center characteristics define the stability windows, redox behavior and functional applicability of tetravalent MOFs in aqueous electrochemical systems.

Although tetravalent MOFs are chemically stable in water and acidic aqueous solutions, it should be noted that they are generally unstable in strongly alkaline solutions.^12,23^ Depending on the type of tetravalent metal and the node connectivity, the maximum pH at which these MOFs can preserve their structural integrity is around 9–12.^12,36^ Thus, their direct use in strongly alkaline electrolytes for electrocatalysis and other electrochemical processes may lead to the degradation of MOFs, followed by the *in situ* formation of MOF-derived materials.^43^ While such MOF-derived materials may sometimes exhibit remarkable and stable electrochemical performance, they should not be identified as MOFs.^16^ In addition to alkaline electrochemical systems, these MOFs are also incompatible with some buffer solutions. Even at neutral pH, the presence of strongly coordinating ions, such as phosphate buffer solutions (PBS) and highly concentrated bicarbonate buffer solutions, can lead to the degradation of such MOFs.^44,45^ For electrochemical reactions requiring buffers, alternatives such as 2-amino-2-(hydroxymethyl)-1,3-propanediol (TRIS), 4-(2-hydroxyethyl)­piperazine-1-ethanesulfonic acid (HEPES), and 3-(N-morpholino)­propanesulfonic acid (MOPS) are more compatible with these MOFs.^44,46^ Therefore, although tetravalent MOFs are usually known as highly stable frameworks, the rational selection of electrochemical applications as well as proper electrolytes for them is still crucial.

Most tetravalent MOFs are intrinsically insulating for electrons.^10^ However, by constructing them with redox-active linkers or incorporating redox-active moieties into them through postsynthetic modifications (PSM),^47^ these MOFs can exhibit redox-based conductivity for electrons at certain applied potentials.^10,48^ Such electronic conductivity coupled with the mass transfer of counterions from the electrolyte, also known as the “redox-hopping phenomenon,” further allows the use of these MOFs as active materials or electrocatalysts for a range of electrochemical applications.^5,49^ On the other hand, even though the MOFs are electrically insulating and redox-innocent, they can act as additives or fillers in nanocomposites to enhance the performance of other active and conductive materials in specific electrochemical processes. These MOFs can also be employed as porous coatings on electrodes or separators to modulate the flux of various ionic species, which is important in both electrocatalysis and batteries. Moreover, MOFs with high ionic conductivity but extremely low electrical conductivity can be incorporated into solid-state or gel electrolytes, which are crucial components in batteries. Structures of some tetravalent MOFs that have been employed in electrocatalysis and electrochemical energy storage, along with their aperture sizes between interconnected pores, are shown in Figure 1. It is important to note that to allow electrochemical reactions to occur within the MOF pores or on the underlying electrode of the MOF coating, the aperture size of the MOF should be sufficiently larger than the sizes of ionic species involved in the reactions.

**Figure 1:**
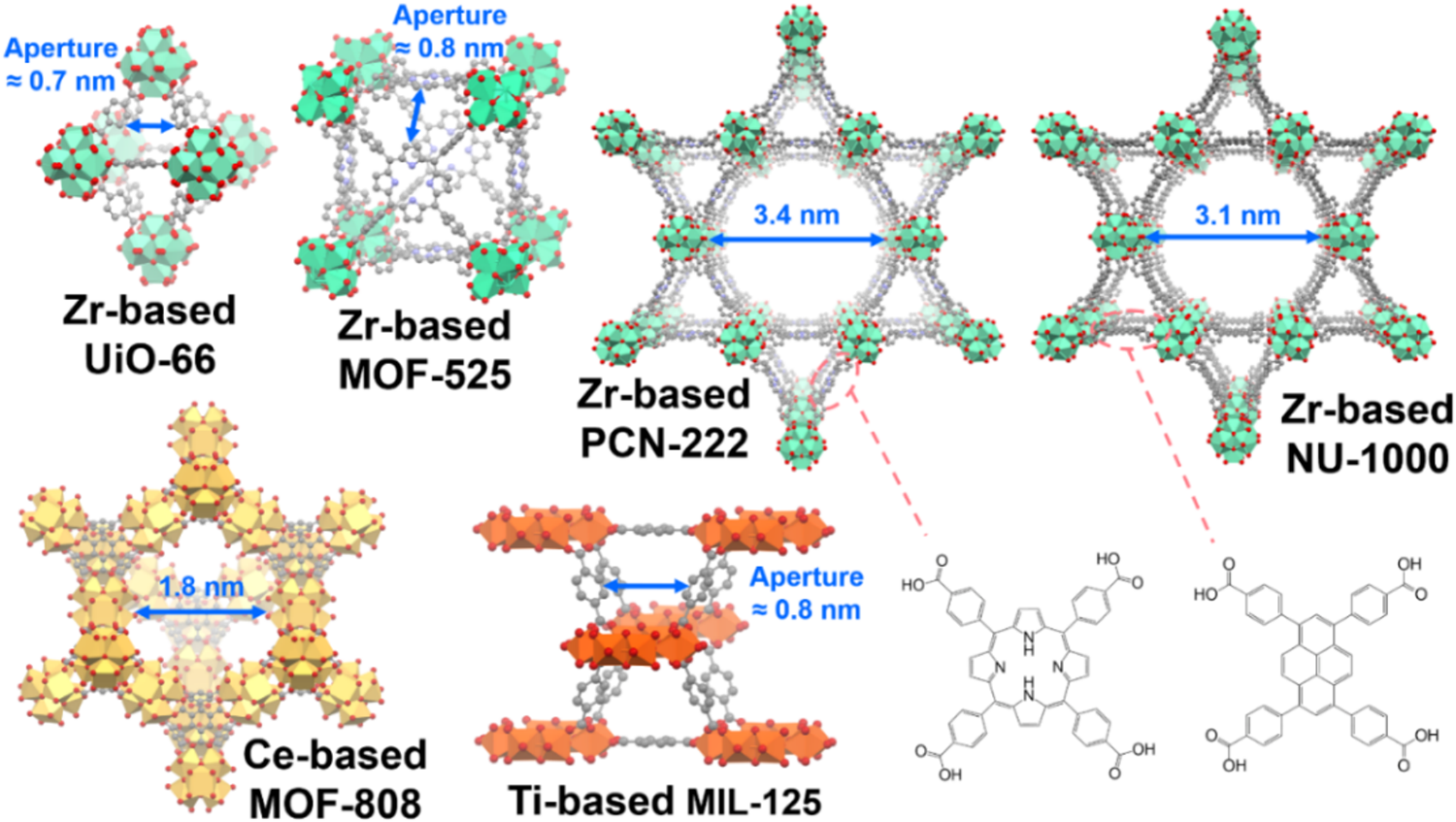
Structures of some tetravalent MOFs commonly used in electrocatalysis and aqueous electrochemical energy storage, highlighting their pore sizes or aperture sizes allowing the penetration of guest molecules. Zr-based MOF-525 and PCN-222 with free-base porphyrinic linkers are shown. Green, yellow, and orange polyhedrons represent zirconium, cerium, and titanium atoms, respectively. Oxygen, carbon, and nitrogen atoms are shown as red, gray, and blue spheres, respectively.

Comprehensive reviews on the application of Zr-MOFs for electrocatalysis, batteries, supercapacitors, and relevant electrochemical applications can be found in our recent reviews.^50,51^ Herein, other tetravalent MOFs, including Ce-MOFs, Ti-MOFs, and Hf-MOFs, and their roles in electrocatalysis and electrochemical energy storage, will be covered as well. Th-MOFs are not included since no study has reported their electrochemical applications yet. In particular, the discussion will mainly focus on electrocatalytic and charge-storage applications with aqueous electrolytes, where these tetravalent MOFs can preserve their structural integrity. The progress and key advances in this subfield, demonstrated by both others and our group, will be highlighted in the following sections.

## 2 Electrocatalysis

Electrochemical reactions can be utilized in numerous energy-conversion processes, including water electrolysis to produce hydrogen energy,^52^ reduction of CO_2_ to produce useful fuels,^53^ and the conversion of nitrogen gas or nitrate ions from wastewater into ammonia.^54^ To reduce the kinetic barrier of each electrochemical reaction, the use of electrocatalysts is usually required. On the other hand, the oxygen reduction reaction (ORR), a crucial half reaction in both fuel cells and air batteries, also requires electrocatalysts to launch at desirable potentials.^55^ In addition, electrocatalysts with selectivity toward specific targeted species can be applied for nonenzymatic sensors.^56^ Thin films of highly stable tetravalent MOFs have thus been widely utilized in these electrocatalytic processes since 2015. Classified by the design strategy and role of MOFs, key advances in this subfield will be overviewed as follows.

### 2.1 Redox-Active MOFs as Electrocatalysts

Serving robust and porous MOFs as supports to immobilize spatially accessible electrocatalytically active sites is a common concept described in the literature. However, almost all tetravalent MOFs are electrically insulating. The reason is that tetravalent MOFs usually contain high-valent d^0^ or f^0^ metal centers that form predominantly ionic M–O­(carboxylate) bonds with hard O-donor ligands, while the organic linkers act as wide band gap π-insulators.^57^ As a result, the frontier orbitals are energetically and spatially separated between metal-based nodes and organic linkers, the orbital overlap along the framework is weak, and the efficient mixed-valence or conjugated pathways for charge conduction are absent.^58,59^ Such facts thus lead to intrinsically low electrical conductivity in typical tetravalent MOFs. In order to render those active sites inside the MOF electrochemically addressable, redox-active moieties uniformly dispersed in the entire framework are necessary to initiate the redox-hopping charge transport.^5,10,48^ These redox-active sites in MOF can also play a role as the electrocatalyst for targeted reactions. To design such redox-active MOFs, feasible strategies include constructing MOFs with redox-active linkers, using the redox activity of nodes, and introducing redox-active moieties through PSMs.

Metalloporphyrin is a well-known unit for electrocatalyzing various reactions, and its activity strongly depends on its metal center. Tetravalent MOFs constructed from porphyrinic linkers were thus widely studied for electrocatalysis. For example, in an early study in 2015, Hupp, Farha, Kubiak, and co-workers reported the redox-based charge transport in Fe-MOF-525, a Zr-MOF constructed from iron–metalated porphyrinic linkers.^60^ With porphyrinic sites as the electrocatalyst, the redox-active MOF thin film could convert CO_2_ into CO in acetonitrile-based electrolytes; see Figure 2a. Subsequent studies by various research groups further investigated the charge-hopping processes and electrocatalytic activity of diverse stable porphyrinic MOFs. Zr-MOFs constructed from iron–metalated or cobalt–metalated porphyrinic linkers could show significant electrocatalytic activity for ORR,^61–63^ while those with nickel–metalated porphyrinic linkers could electrocatalyze oxygen evolution reaction (OER),^64^ a crucial half reaction of water electrolysis. A recent study also revealed that thin films of a Zr-MOF constructed from redox-active manganese–metalated porphyrinic linkers could electrocatalyze the reactions of various environmental analytes, which is desirable for selective sensing.^65^ In addition to Zr-MOFs, in 2018, Lin et al. also demonstrated the use of a Hf-MOF constructed from cobalt–metalated porphyrinic linkers for HER in acidic aqueous solutions.^66^

**Figure 2:**
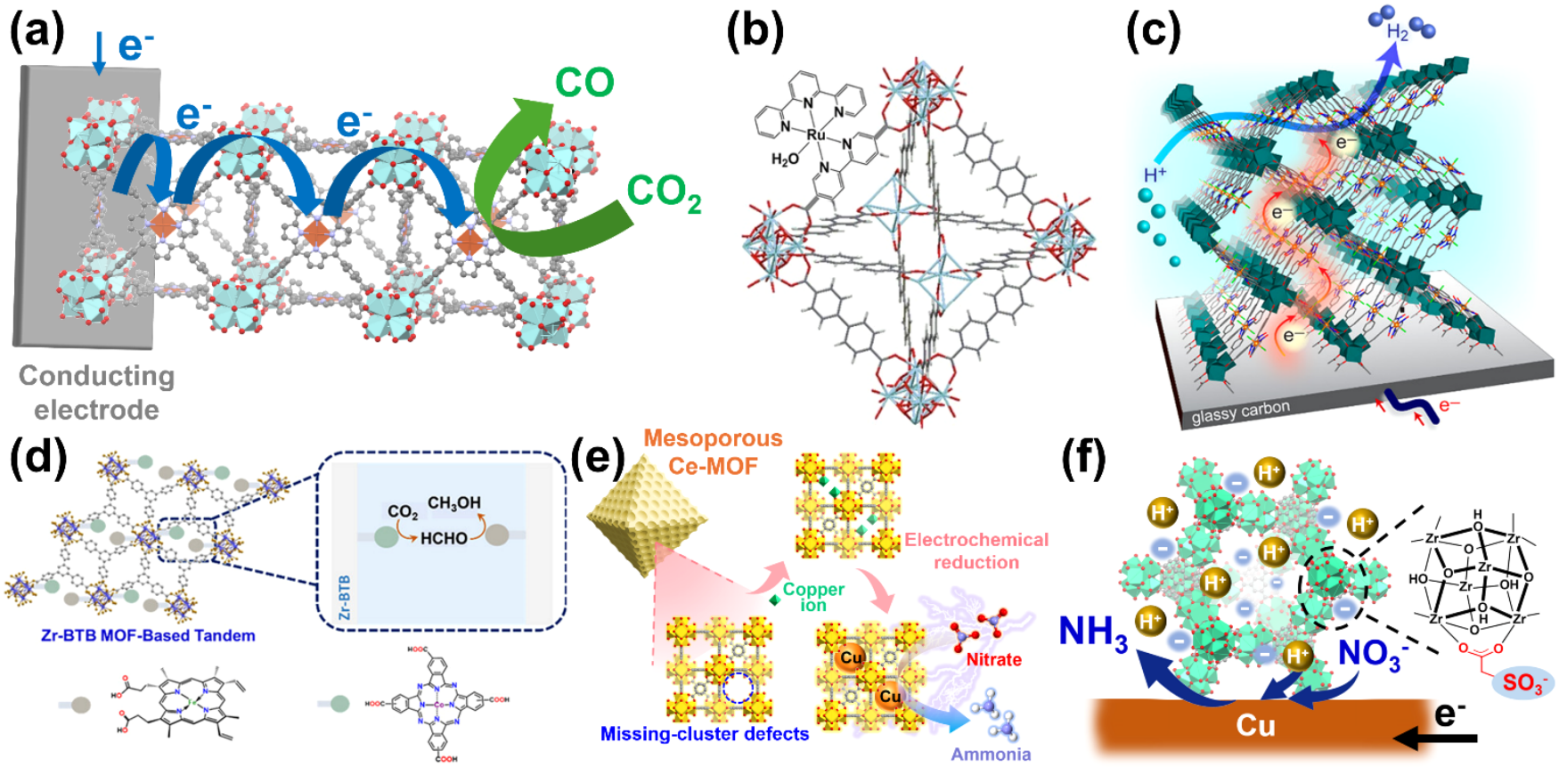
Redox-active tetravalent MOFs for electrocatalytic (a) CO_2_ reduction to CO,^60^ (b) OER (Copyright 2017 John Wiley and Sons.),^67^ (c) HER (Copyright 2019 American Chemical Society),^70^ and (d) cascade conversion from CO_2_ to methanol (Copyright 2025 under CC-BY 4.0).^81^ (e) Pore-confined Cu NPs in the Ce-MOF with large mesopores for NO_3_RR.^93^ (f) SO_3_-MOF-808 coating to boost the selectivity of NO_3_RR toward ammonia occurring on the underlying copper-based electrocatalyst (Copyright 2024 under CC-BY 4.0).^97^

In addition to porphyrins, utilizing other redox-active and catalytically active linkers to design tetravalent MOFs is also feasible. For example, in 2017, Morris et al. reported the redox-hopping behavior in a Zr-MOF constructed from linkers with ruthenium-based moieties, and this MOF was found capable of electrocatalyzing OER in neutral MOPS-based buffer solutions (Figure 2b).^67,68^ The similar Zr-MOF with ruthenium-based moieties could also catalyze OER in borate buffer solutions at pH = 8.4, as demonstrated by Ott and co-workers.^69^ Another noticeable example was also reported by Ott et al., who designed UU-100­(Co), a Zr-MOF constructed from redox-active and electrocatalytic cobaloxime-based linkers (Figure 2c).^70^ The MOF could electrocatalyze hydrogen evolution reaction (HER) in acetate buffer solutions at pH = 4 while preserving its structural integrity.

With potential redox activity from their nodes, stable Ce-MOFs and Ti-MOFs were also applied for electrocatalysis in a few studies. In 2022, we first demonstrated the use of a stable cluster-based Ce-MOF for electrocatalysis.^71^ A Ce-MOF, Ce-MOF-808, was found to show reversible electrochemical activity between Ce­(III) and Ce­(IV) in neutral MOPS buffer solutions. The electrochemical formation of Ce­(III) species could catalyze the reduction of dopaquinone through the EC’ pathway, which could be further employed in reductive sensing of dopamine (DA) to avoid interference. In addition to utilizing the redox activity of cerium-based nodes, redox-active and catalytically active ruthenium-based moieties could also be incorporated into Ce-MOFs as a proportion of linkers, as demonstrated by Pushkar et al. in 2024; the resulting Ce-MOF was applied for photoelectrochemical OER in acidic aqueous solutions.^72^ For Ti-MOFs, owing to their limited structural diversity, examples of using them in electrocatalysis are still rare in the literature. One noticeable work was reported by Lu, Xu and colleagues in 2024, demonstrating the use of a Ti-MOF constructed from 2,5-dihydroxyterephthalic acid linkers for the electrocatalytic reduction of both nitrate and CO_2_ to yield urea in neutral aqueous solutions.^73^ The titanium nodes of the MOF were reported as active sites for the adsorption of both nitrate and CO_2_ during the electrocatalysis.

Immobilizing spatially dispersed active sites in MOFs through PSMs is another strategy to design redox-active and electrocatalytic tetravalent MOFs. For example, in an early study reported by Hupp, Farha, and co-workers, cobalt­(II) ions were postsynthetically installed on the hexa-zirconium nodes of a Zr-MOF, NU-1000.^74^ With the charge hopping between redox-active cobalt sites as well as the catalytic activity of cobalt, the MOF thin film could electrocatalyze OER in weakly alkaline electrolytes at around pH = 11. On the other hand, since molybdenum sulfides (MoS_*x*_) are well-known electrocatalysts for HER in acidic electrolytes, the immobilization of spatially dispersed MoS_*x*_ clusters in a Zr-MOF could result in a redox-active MOF capable of electrocatalyzing HER.^75^ Recently, we employed PSM to immobilize redox-active and spatially dispersed iridium ions onto the nodes of a series of Zr-MOFs.^76^ Owing to the catalytic activity of iridium for OER in acidic aqueous electrolytes, where these MOFs are chemically stable, the resulting MOFs could exhibit electrocatalytic activity for OER in 0.1 M HClO_4_ aqueous solutions. The rigid MOFs with sufficiently large pores can also serve as supports for redox-active polyoxometalates (POMs). For example, in our previous study in 2020, a redox-active vanadium-based POM was immobilized in a Zr-MOF to render redox-based conductivity, and the resulting MOF could be employed as the electrocatalyst for DA oxidation.^77^ Loading the POM into a redox-active porphyrinic MOF could further enhance the electrocatalytic activity of the MOF for CO_2_ reduction, as demonstrated by Ma, Lan, He, and co-workers.^78^ In addition to redox-active metal ions or clusters, redox-active organic moieties can also be immobilized in MOFs through PSMs. For example, by immobilizing metalloporphyrin molecules in a redox-innocent Zr-MOF, the framework could become redox-active, electrochemically addressable, and catalytically active for ORR.^79^ A similar strategy was also demonstrated in another recent work by Deria et al., showing the immobilization of redox-active cobalt–metalated phthalocyanine into a Zr-MOF for electrocatalytic CO_2_ reduction.^80^ Very recently, Hod et al. further extended this concept to the simultaneous immobilization of two different redox-active ligands, cobalt–metalated phthalocyanine and iron–metalated porphyrin, onto the nodes of a two-dimensional (2D) Zr-MOF, Zr-BTB.^81^ In the presence of dual active sites, the resulting redox-active Zr-MOF could electrocatalyze the cascade conversion from CO_2_ to methanol with formaldehyde as the intermediate; see Figure 2d.

### 2.2 Catalytically Inactive MOFs in Composites

Even without the redox and electrocatalytic activity, stable and porous tetravalent MOFs may play a role in nanocomposites to boost the performance of other electrocatalysts. The MOF may adjust the microenvironment and change the intermediate adsorbed on the neighboring catalyst surface.^50^ Furthermore, it may also prevent the catalytic material present in the composite from aggregation.^50^

This concept was demonstrated by Hupp, Farha, and coauthors in an early work in 2015.^82^ By electrodepositing nickel sulfide into a Zr-MOF thin film, the electrocatalyst for HER in acidic electrolytes was prepared, and the MOF with proton-conducting characteristics was found to accelerate the HER occurring on the neighboring nickel-sulfide catalyst. Similarly, the composite composed of a Zr-MOF and MoS_*x*_ was reported to boost the activity of MoS_*x*_ for HER.^83^ The nanocomposite with 2D Zr-MOF molecular sheets to disperse the electrocatalyst, Bi_2_O_3_ nanowires, could also facilitate the conversion of CO_2_ to formate.^84^ In our previous work, we also found that by electroplating metallic cobalt into a Zr-MOF thin film, the obtained cobalt could exhibit much better electrocatalytic activity for H_2_O_2_ oxidation compared to the MOF-free cobalt.^85^

Using the robust MOF thin film to confine catalytic metallic nanoparticles (NPs) near the electrode surface can prevent these NPs from agglomeration, which is an effective route of employing stable MOFs in electrocatalysis, even though the frameworks are not conductive nor catalytically active. In 2017, Hupp, Farha, and co-workers first demonstrated this concept by immobilizing Cu^2+^ ions onto the nodes of a Zr-MOF thin film, followed by the electrochemical reduction of a part of the copper sites into pore-confined Cu NPs.^86^ Such Cu NPs located near the underlying electrode could exhibit electrocatalytic activity for CO_2_ reduction to produce formate and CO in NaClO_4_-based aqueous solutions. Recent studies further developed such Zr-MOF-confined metallic NPs for diverse electrocatalytic processes, including Cu NPs@MOF for HER,^87^ bimetallic Pd/Cu NPs@MOF for nitrogen reduction,^88^ Pd NPs@MOF for nitrate reduction reaction (NO_3_RR) to produce ammonia,^89^ and Zn/Cu NPs@MOF for NO_3_RR.^90^ Our previous work also showed that the pore-confined Ag NPs in a Zr-MOF could electrocatalyze the oxidation of nitrite ions for electrochemical sensing purposes.^91^ It is worth mentioning that, in addition to preventing these surface NPs from agglomeration, in some cases, functional groups on the nodes or linkers of the MOF can also affect the adsorption of intermediates occurring on the neighboring NPs.^90^ Modulating the functional groups in the MOF host thus becomes another key to enhancing the activity and selectivity of the pore-confined catalytic NPs.

Ce-MOFs were also employed as similar porous hosts for electrocatalytically active pore-confined NPs. For example, in 2022, Cao et al. performed the immobilization of copper ions in a Ce-MOF, followed by the electrochemical reduction to form small Cu NPs confined in the Ce-MOF.^92^ These Cu NPs were found to exhibit remarkable electrocatalytic activity for NO_3_RR. In our very recent work, we further extended this concept to the Ce-MOF with large mesopores and hierarchical porosity, as shown in Figure 2e.^93^ We found that by employing the Ce-MOF with large mesopores (around 10 nm) created by soft templates, the resulting pore-confined Cu NPs could achieve a much faster reaction rate and a higher Faraday efficiency toward ammonia during NO_3_RR under neutral pH. This enhancement was found more obvious at large overpotentials, which is attributed to the faster mass transfer of nitrate ions in large mesopores compared to that in structure-derived micropores. Large mesopores and hierarchical porosity are thus another crucial consideration for the design of MOF-based electrocatalysts.

### 2.3 MOFs as Porous Coatings

Stable and porous MOFs can also be employed as membrane-type coatings on top of the electrocatalytic electrodes to modulate the local concentrations of various ionic reactants and thus adjust the selectivity between multiple reactions. With this strategy, the electrical conductivity and catalytic activity of the MOF are no longer necessary, and the design and synthesis of the functional MOF and the state-of-the-art electrocatalyst can be fully decoupled.

In 2021, Hod et al. demonstrated this concept by coating a membrane of a Zr-MOF onto the silver electrode, a well-known electrocatalyst for CO_2_ reduction.^94^ With the positively charged trimethylammonium groups incorporated within the MOF, the porous coating served as an ion-gating membrane to retard HER, resulting in an enhanced Faraday efficiency for CO_2_-to-CO conversion occurring on the underlying silver surface. Very recently, the same group also extended this concept by coating a Zr-MOF thin film on top of the bismuth electrode, which is capable of electrocatalyzing the oxidation of benzyl alcohol.^95^ It was found that the MOF coating could enrich hydroxide species and stabilize the desirable product, leading to an enhanced selectivity compared to that achieved by the bare bismuth electrode.

In 2023, we introduced the concept of stable and porous MOF coatings to electrochemical sensing applications.^96^ Thin film of a Zr-MOF with postsynthetically immobilized sulfonate-based ligands on its nodes, SO_3_-MOF-808, was coated on top of the graphene-based electrode that is capable of electrocatalyzing the oxidation of DA. With the negatively charged sulfonate groups to preconcentrate the positively charged DA and repulse other anionic interfering species, the selectivity of the electrode for DA sensing could be enhanced with the help of the porous MOF coating. This concept was further extended to electrocatalytic NO_3_RR in neutral aqueous electrolytes, as demonstrated by our recent work (Figure 2f).^97^ With the SO_3_-MOF-808 coating to serve as a “proton sink” near the underlying copper-based electrocatalyst, the nine-proton-coupled production of ammonia from nitrate can be facilitated compared to the two-proton-coupled nitrite formation, leading to a better selectivity of NO_3_RR toward ammonia. Such porous and stable MOF coatings are expected to boost the performance of diverse electrocatalysts for other proton-coupled processes.

In this section, we classify the tetravalent MOF-based materials used in electrocatalytic applications into three categories, including redox-active MOFs, electrocatalytically inactive MOFs in composites, and porous MOF coatings. All of them take advantage of the interconnected porosity and structural robustness of tetravalent MOFs. With the redox activity in the entire framework to render charge hopping, these materials can function as stable host matrices for atomically dispersed electrocatalytically active sites. Despite these advantages, several common bottlenecks persist in such MOF-based electrocatalysts, including low electrical conductivity, incomplete understanding of solid–liquid interfacial processes, and framework instability under harsh operating conditions containing strongly coordinating ions in electrolytes.^12,44,50,59^ The issue of low conductivity may be partially resolved by designing MOF-based composites with other conductive materials, but it should be noticed that, the electronic conduction in the MOF loaded with active sites, rather than that solely in the conductive material, is necessary to make internal active sites electrochemically addressable. In addition to enhancing conductivity of the framework, reducing the particle size of redox-active MOF with the use of another conductive matrix in the composite should be a feasible solution to achieve high performance in electrocatalysis. On the other hand, with the use of functional tetravalent MOFs as porous coatings on top of other electrocatalysts, the electronic conduction in MOF is no longer required, allowing more flexible selection and design of both MOFs and electrocatalytic materials for certain reactions.

## 3 Supercapacitors

Supercapacitors are fast-charging devices bridging the gap between batteries and conventional capacitors.^98^ As one of the most common active materials for supercapacitors, carbon relies on the non-Faradaic process and the corresponding electrical double layer to store charges.^98^ On the other hand, pseudocapacitive materials, such as manganese oxide, ruthenium oxide, cobalt oxide, and polyaniline, utilize their facile Faradaic reactions to achieve high specific capacitances.^98^ Organic electrolytes are typically used in double-layer capacitors to achieve a wide voltage window, while aqueous electrolytes at certain pH levels are usually required for the electrochemical reactions of pseudocapacitive materials. Owing to the insulating nature of tetravalent MOFs, it is very challenging to employ their pristine versions in supercapacitors to reach a high specific capacitance and fulfill the fast-charging criteria. Designing nanocomposites consisting of such stable MOFs and other conductive materials is thus generally required. In addition, several studies have attempted to use tetravalent MOFs in strongly alkaline electrolytes to achieve superior capacitive performance. However, since these MOFs can undergo quick degradation in such electrolytes,^12,23^ these materials for supercapacitors should be realized as metal hydroxides or their composites with organic moieties derived from MOFs.

Composites with redox-active MOFs and nanocarbons are common candidates for supercapacitors. In 2014, Yaghi, Kang, and co-workers pioneered the use of MOFs in supercapacitors, utilizing nanocrystals of twenty-three distinct MOFs, including some tetravalent MOFs, in symmetric supercapacitors with organic electrolytes.^99^ With graphene as the conductive binder between MOF nanocrystals, the capacitor with a Zr-MOF constructed from 2,2-bipyridine-5,5-dicarboxylate linkers, MOF-867, could exhibit a stack capacitance of 0.644 F/cm^3^ and an area capacitance of 5.085 mF/cm^2^. Subsequent studies by various researchers have developed various composites composed of nanocarbons and tetravalent MOFs for aqueous supercapacitors.^100–102^ For example, our early work showed that by growing defective UiO-66 nanocrystals on dispersed carboxylic-functionalized carbon nanotubes (CNTs), followed by the PSM to immobilize manganese sites in MOF crystals, the obtained conductive and redox-active nanocomposites could be applied for aqueous supercapacitors in neutral aqueous electrolytes.^46,100^ Recently, Fischer, Jayaramulu, Zbořil, Dubal, and co-workers demonstrated the growth of a redox-active Zr-MOF, UiO-66-NH_2_, between the fully dispersed carboxylate-functionalized graphene.^102^ The MOF nanocrystals with amino groups on their external surface could form covalent bonds to bridge 2D graphene sheets, leading to a three-dimensional (3D) network, as illustrated in Figure 3a. With the electrical conductivity of the graphene and the pseudocapacitance of the MOF, the nanocomposite could achieve a specific capacitance of 651 F/g in a Na_2_SO_4_-based aqueous electrolyte.

**Figure 3:**
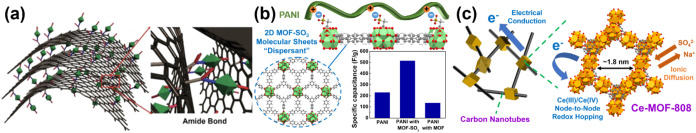
(a) 3D network with redox-active Zr-MOF nanocrystals and graphene sheets for aqueous supercapacitors, reprinted from ref 102 under CC-BY 4.0. (b) 2D Zr-MOF molecular sheets as the dispersant for PANI and the corresponding capacitive performance in HCl-based acidic solutions, reprinted from ref 106 with permission. Copyright 2023 American Chemical Society. (c) Aqueous electrochemistry of Ce-MOF and the use of Ce-MOF-CNT nanocomposites for aqueous supercapacitors, reprinted from ref 33 with permission. Copyright 2021 American Chemical Society.

Conducting polymers are well-known materials for supercapacitors. Among them, polyaniline (PANI) is especially of interest owing to its high specific capacitance, originating from its strong redox activity in acidic electrolytes.^98^ Since tetravalent MOFs are generally stable in acids, nanocomposites consisting of such MOFs and PANI are highly attractive for supercapacitors. In 2018, Shao et al. reported an early example of such nanocomposites for supercapacitors.^103^ Aniline monomers could be polymerized in HCl aqueous solutions by serving ammonium persulfate (APS) as the initiator, and the Zr-based UiO-66 crystals could be employed as the additive during such oxidative polymerization to obtain MOF-PANI nanocomposites, with the crystallinity of MOF preserved. Such composites could thus be utilized as active materials for supercapacitors in acidic aqueous electrolytes. Other researchers have also demonstrated such *in situ* polymerization in the presence of dispersed porous MOF crystals to synthesize MOF-PANI composites, aiming for the use in supercapacitors.^104,105^ In 2023, we attempted to extend this MOF-PANI design from 3D MOF crystals to 2D dispersible MOF sheets, also known as metal–organic layers, for the first time.^106^ Compared to 3D MOFs, 2D MOFs can be dispersed as molecular sheets in the solution for polymerization; the diffusion of both aniline monomer and APS should thus be more facile between dispersed 2D sheets compared to that within the 3D framework. Dispersed 2D sheets of Zr-BTB were used as the additive during the polymerization of aniline. Furthermore, negatively charged sulfonate-based ligands were further immobilized on the 2D MOF sheets through PSM to synthesize the anionic 2D MOF. As illustrated in Figure 3b, with the negatively charged MOF sheets to attract and align aniline monomers during the *in situ* polymerization, the resulting MOF-PANI nanocomposite could achieve outperforming capacitive performance in HCl-based aqueous electrolytes compared to both the pristine PANI and the PANI with nonfunctionalized Zr-BTB.

Ce-MOFs with redox activity could also act as pseudocapacitive materials for aqueous electrochemical energy storage. But rather than using Ce-MOFs, early attempts in the literature mostly employed ceria derived from MOFs, either prepared by high-temperature treatments or exposure to strongly alkaline solutions. In 2021, we reported the aqueous electrochemistry of Ce-MOFs and the corresponding redox-hopping behavior for the first time.^33^ As shown in Figure 3c, a minor proportion of cerium atoms in hexa-cerium nodes of the Ce-MOF, Ce-MOF-808, could be redox-active between Ce­(IV) and Ce­(III). Redox-based charge transport could thus occur within a limited portion of the MOF crystal, rendering this MOF electrochemically active. By further growing Ce-MOF nanocrystals on CNTs, the obtained nanocomposites could exhibit better capacitive performance compared to both the pristine CNTs and pristine MOF in neutral Na_2_SO_4_-based aqueous electrolytes. The redox-active Ce-MOF thin film could also be subjected to electropolymerization to deposit a conducting polymer, poly­(3,4-ethylenedioxythiophene) (PEDOT), within its pores, as demonstrated in our recent work.^107^ The pseudocapacitance of the Ce-MOF in aqueous electrolytes could thus be largely amplified in the presence of PEDOT. It is important to notice that the electrochemical processes of Ce-MOF and the mechanism hidden behind them were probed by Noh et al. in a recent study.^35^ Findings showed that in neutral and weakly alkaline aqueous electrolytes, where the Ce-MOF is stable, the electrochemical process is proton-coupled, with the protons on hexa-cerium nodes involved.

Ti-MOFs have attracted increasing attention owing to the intrinsic advantages of titanium, including its high natural abundance in the earth’s crust, low toxicity, and unique photoredox activity.^108^ Nevertheless, Ti-MOFs remain relatively scarce compared with other tetravalent systems, as their rational design and controlled synthesis are intrinsically challenging due to the high oxophilicity and complex coordination chemistry of titanium.^40^ Consequently, the direct use of prototypical Ti-MOFs such as MIL-125­(Ti) and NH_2_-MIL-125­(Ti) in electrochemical energy storage is still barely reported so far. Because MIL-125­(Ti) is structurally related to TiO_2_, it has been more widely explored for photocatalysis rather than for electrochemical applications, despite sharing the high chemical robustness that is the characteristic of tetravalent MOFs.

Compared to using pristine Ti-MOFs, an alternative strategy is to employ MIL-125­(Ti) as a precursor or structural template to generate TiO_2_ or TiO_2_/C-based derivatives, which exhibit enhanced redox activity and electrical conductivity and have been successfully applied as electrode materials for supercapacitors.^109^ A representative example is the study by Manyala et al. in 2025,^110^ demonstrating the direct use of bimetallic MIL-125­(Ti, Mn) as the material for supercapacitors in 6 M KOH. While the MOFs should be converted into MOF-derived oxides in such electrolytes, the improved specific capacitance and rate performance could be achieved owing to the 3D porous network and the synergistic contribution of Ti/Mn dual redox-active sites.

It is worth noting that few studies have reported the direct application of Hf-MOFs in supercapacitors. In contrast, a considerable number of investigations have focused on proton conduction, which is highly related to electrochemical devices and consistently demonstrates the outstanding water stability and structural robustness of Hf-MOFs.^111^ Although Zr-MOFs currently dominate the literature landscape in electrochemical research, recent developments in Ti-based and Ce-based systems, together with the early exploratory work on Hf-MOFs, collectively indicate that tetravalent MOFs hold broad and evolving potential across diverse platforms of aqueous electrochemical energy storage.

From a mechanistic perspective, the charge-storage behavior in tetravalent MOFs is largely governed by their framework stability, pore accessibility for counterions, efficient electronic conduction in the framework, and the nature of redox-active centers. These MOF-based systems face several common bottlenecks, including intrinsically low electrical conductivity, limited pseudocapacitive activity in pristine frameworks, and insufficient understanding of how pore structure and defect chemistry affect ion adsorption and charge-storage mechanisms.^112^ In particular, unlike batteries, supercapacitors require fast charging and discharging processes, which means that as an active material, facile electronic conduction and fast mass transfer of ions in the MOF are both required. The former is especially challenging for almost all tetravalent MOFs. Designing composite materials with conducting polymers or carbons as the major conductive phase, along with the stable MOFs to provide interconnected porosity for ionic transport and/or redox-active sites for pseudocapacitance, is thus a rational strategy for designing high-performance active materials in supercapacitors.^33,51^

## 4 Aqueous Batteries

As the most commonly reported type of aqueous batteries, rechargeable zinc-ion batteries (ZIBs) have attracted great attention as a safer and cheaper alternative to nonaqueous batteries such as lithium-ion batteries.^113^ Another noticeable type of aqueous batteries belongs to vanadium flow batteries (VFBs), which are commonly used for large-scale electrochemical energy-storage systems.^114^ Both ZIBs and VFBs require aqueous electrolytes containing high concentrations of metal salts. In addition, ZIBs usually require slightly acidic electrolytes, while electrolytes with strong acids, such as sulfuric acid (H_2_SO_4_), are usually used in VFBs. Since most MOFs are not chemically stable in such acidic aqueous environments, it is fairly challenging to apply most pristine MOFs for these aqueous batteries. It is thus more common to see the use of MOF-derived materials in ZIBs and VFBs in early studies. Tetravalent MOFs, which are highly stable in acids, have thus become the unique category of MOFs that are highly appealing for ZIBs and VFBs. The application of tetravalent MOFs in these aqueous batteries belongs to a recently emerging subfield, and most studies on this topic were published in the past five years. Zr-MOFs were mostly utilized, and the use of a Ti-MOF in ZIBs was also demonstrated. But to date, the use of other tetravalent MOFs such as Hf-MOFs and Ce-MOFs in aqueous batteries has not been reported.

### 4.1 MOFs on Electrodes

In a ZIB, a metallic zinc foil acts as its negative electrode, and a redox-active transition metal oxide is usually employed as the material on the positive electrode. But to date, the use of tetravalent MOFs on positive electrodes of ZIBs has barely been explored yet. In most studies, such MOFs were utilized as porous coatings on top of negative electrodes of ZIBs to suppress the growth of zinc dendrites during the long-term charge–discharge process.

For example, Nam and coauthors demonstrated the use of a tetravalent MOF on negative electrodes of ZIBs for the first time in 2022.^115^ A composite coating containing crystals of UiO-66-(COOH)_2_, an analog of Zr-based UiO-66, was deposited on top of the zinc electrode of the ZIB. The MOF coating was found to regulate the flux of zinc ions, which could suppress the formation of zinc dendrites. Subsequent studies reported by other groups also employed various Zr-based UiO-66 analogs containing various functional groups as coatings on negative electrodes of ZIBs for suppressing dendrites.^116,117^ One recently published and noticeable work in this subfield was reported by Zhou, Pan, Chang and co-workers, demonstrating the use of two Zr-MOFs with different pore sizes, MOF-808 and MOF-801, as protecting coatings on negative electrodes of ZIBs.^118^ The authors found that the enriched carboxyl groups in the pores of these MOFs could act as “water catchers” to promote the desolvation of zinc ions, and the MOF with smaller pores, MOF-801, could play a better role in desolvating zinc ions compared to the MOF with larger pores. MOF coatings with smaller pore sizes were thus considered as better candidates for negative electrodes of ZIBs. But it should be noted that published studies on such MOF coatings for ZIBs are still quite limited with the use of only a few types of Zr-MOFs. Effects of the node connectivity, topology, and type of metal ions in the cluster on the resulting performance of such MOF coatings in ZIBs, have not been explored yet.

In addition to Zr-MOFs, in 2023, Han, Li, Wang, and coauthors demonstrated the use of a Ti-MOF as coatings on negative electrodes of ZIBs for the first time.^119^ As shown in Figure 4a, a Ti-MOF with small pore sizes of around 0.5–0.7 nm, NH_2_-MIL-125, was employed as an ion-sieving coating on top of zinc electrodes. It was found that the Ti-MOF coating could not only render more uniform deposition of zinc on the electrode, but also suppress the HER, leading to a much better cycling stability of the resulting ZIBs.

**Figure 4:**
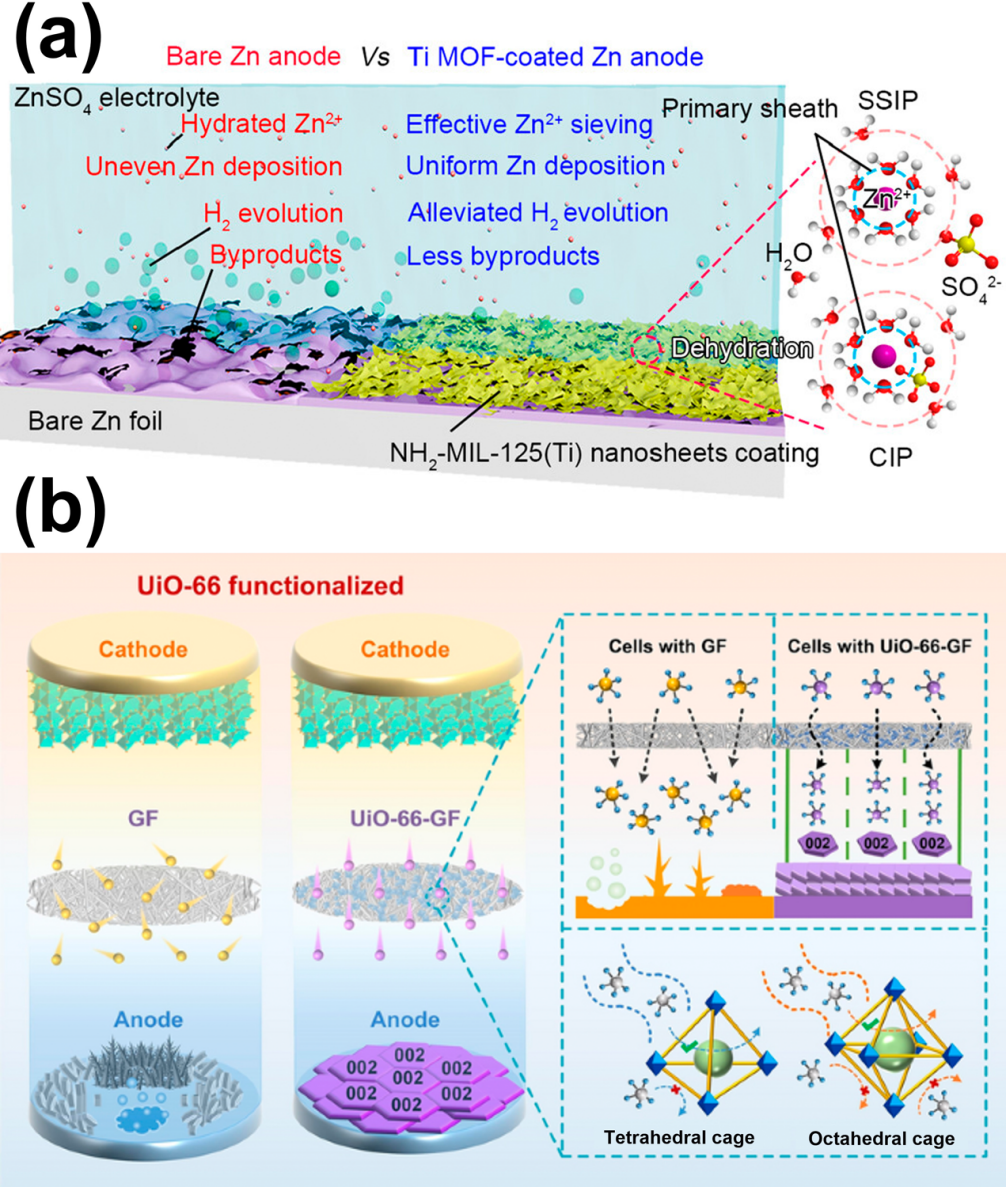
(a) Ti-MOF as the coating on top of the negative electrode for the use in ZIBs, reprinted from ref 119 with permission. Copyright 2023 American Chemical Society. (b) Zr-MOF incorporated in membrane separators of ZIBs to suppress the growth of dendrites, reprinted from ref 120 under CC-BY 4.0.

### 4.2 MOFs in Separators

Compared to electrodes, these highly stable and electrically insulating MOFs were more commonly utilized in separators of aqueous batteries. For example, by coating or blending MOF crystals with regular porosity and designed functional groups into the separator of a ZIB, the ionic flux of zinc ions may be regulated, which is expected to suppress the formation of dendrites on the zinc electrode. On the other hand, the membrane separator in a VFB requires a high proton conductivity but a low permeability of vanadium-based redox-active ions in the aqueous electrolyte containing high concentrations of both strong acid and vanadium ions.^114^ Water-stable MOFs with small micropores that can block the penetration of large vanadium-based ions thus become ideal candidates for the use in such separators. But to date, all examples in this subfield have only used Zr-MOFs; the application of other tetravalent MOFs in separators of aqueous batteries has not been explored yet.

For example, an early study reported by He, Zhou, and colleagues in 2022 demonstrated the direct growth of UiO-66 crystals on glass fiber separators of ZIBs.^120^ As shown in Figure 4b, in an aqueous electrolyte containing concentrated zinc sulfate, the porous MOF coating could regulate the flux of zinc ions, resulting in the preferred deposition of zinc with the (002) plane on the negative electrode and thus suppressing the formation of zinc dendrites. The authors also found that the generated (002) plane of zinc could further suppress HER, which is also effective in enhancing the long-term stability of the battery. Similar incorporations of stable MOFs were also demonstrated in subsequent studies, with the use of functionalized Zr-MOFs, e.g., UiO-66 with amino groups on its linkers and that with sulfonic acid on its linkers.^121,122^ This strategy reveals that even though the MOF is not electrochemically active nor deposited on the electrode surface, its presence in the membrane can modulate the electrochemical reactions occurring on the electrode surface.

In addition to ZIBs, such water-stable MOFs can also be employed in separators of VFBs to suppress the crossover penetration of electrolytes on both sides. This idea was first reported by Zhang, Wang, and co-workers in 2017, demonstrating the incorporation of a small amount of UiO-66-based MOF crystals into poly­(ether ether ketone)-based membranes of VFBs.^123^ It was found that the MOF additive with small micropores could enable the sieving effect to suppress the permeability of VO^2+^ ions through the membrane while preserving the similar proton conductivity; a better long-cycle stability of VFBs could thus be achieved. In addition to the sieving effect, functional groups in the MOF pores can also play a role in the separators of VFBs, by either promoting the mass transfer of protons or suppressing the penetration of vanadium-based ions.^124,125^ One recent example was reported by Wang et al. in 2024, demonstrating the PSM to graft ethylenediaminetetraacetic acid on the nodes of MOF-808.^126^ With this MOF as the additive in separators of VFBs, the terminal carboxylic groups could largely promote proton conductivity while suppressing the mass transfer of vanadium-based ions across the membrane. It is worth mentioning that in most VFBs, strongly acidic aqueous electrolytes containing 3 M of H_2_SO_4_ and vanadium-based ions were used, and it was reported that Zr-MOFs such as MOF-808 and MOF-801 could preserve their crystallinity after the exposure to such acidic solutions for a few days.^124^ Such exceptional chemical stability in strong acids, which is hardly achieved by most MOFs, thus makes these tetravalent MOFs unique and attractive candidates for use in VFBs.

In summary, owing to their exceptional stability in water, tetravalent MOFs have been employed as active materials on electrodes or additives in separators in aqueous batteries. But current studies remain limited, mostly only focused on the use of Zr-MOFs. The use of Hf-MOFs, Ce-MOFs and Th-MOFs in these aqueous batteries has not been explored yet. In addition, since existing studies are still limited, even for Zr-MOFs, the effects of their structure, pore size and chemical functionality on the resulting performance of ZIBs or VFBs have still been barely explored. From a mechanistic perspective, the interaction between redox-active metal ions and the MOF hosts, the change of local coordination environments induced by MOFs, and the role of defects in MOFs, remain insufficiently understood in both ZIBs and VFBs. For VFBs where strongly acidic electrolytes are needed, although a few studies have reported the stability of selected Zr-MOFs under such harsh conditions,^124^ the stability and applicability of other tetravalent MOFs in such systems, without fully deconstructing the framework, are still unclear. These facts thus provide several opportunities for fundamental studies in this subfield, aiming for enhanced energy-storage performance.

## 5 Summary and Outlooks

With chemical stability in water, relatively high specific surface area, and highly tunable pore structures and chemical functionality, tetravalent MOFs including Zr-MOFs, Ce-MOFs, Ti-MOFs, Hf-MOFs and Th-MOFs are highly attractive for electrocatalysis and aqueous electrochemical energy storage. Over the past ten years, some of these MOFs have been widely employed in electrocatalysis including HER, OER, ORR, CO_2_ conversion and ammonia production, catalytic electrochemical sensors for aqueous samples, aqueous supercapacitors, and aqueous batteries such as ZIBs and VFBs.

### 5.1 Electrochemically Active MOFs

The interconnected porosity and high specific surface area of MOFs render them attractive candidates to support spatially dispersed and highly accessible electrochemically active sites. However, it should be noticed that as an electrochemically active material in every aforementioned application, the electronic conduction within the selected MOF must be considered to make the internal active sites electrochemically addressable. Otherwise, only the external surface of MOF crystals in contact with the underlying electrode can be electrochemically active. Redox conductivity, also known as the redox-hopping phenomenon, is thus usually used to create charge-transport pathways in these stable and porous MOFs, as introduced in Section 2.1. For frameworks with redox-innocent nodes such as Zr-MOFs and Hf-MOFs, redox-active sites can be incorporated into these MOFs, by either introducing the redox-active linkers or performing the PSM to immobilize redox-active moieties. Ce-MOFs and Ti-MOFs possess redox activity originating from their metal nodes, and the redox activity of these metal nodes was found electrochemically addressable in aqueous media.^27,33^ These redox-active and stable MOFs can further be incorporated with conductive supports, such as carbons or conducting polymers, to design composites with facilitated electronic conduction between adjacent MOF crystals. All these characteristics provide lots of opportunities in designing and utilizing such MOF-based materials for various electrochemical applications. But to date, most reported redox-active tetravalent MOFs and their composites are still under the scope of Zr-MOFs. The electrochemical behaviors and corresponding applications of structurally diverse redox-active Ce-MOFs, Hf-MOFs and Ti-MOFs, and the design of their electrochemically active composites, have only been explored in limited studies. Several opportunities are still there in both fundamental studies and application-oriented research in this subfield. In addition, most reported redox-active tetravalent MOFs were applied for electrocatalysis. We thus believe that these MOFs should be highly attractive for redox-based electrochemical sensing of environmental species. With the rational selection of redox-active moieties, they could also act as active materials on positive electrodes of aqueous batteries such as ZIBs, where a redox-active material is required. Examples of these directions are still rare in the literature.

### 5.2 Electrochemically Inactive MOFs and Their Diverse Roles

Without the electrical conductivity and redox activity, such highly stable MOFs can also serve as additives or fillers in other conductive materials to further enhance their electrochemical performances. For this strategy, most published studies reported nanocomposites composed of MOFs and conducting polymers such as PANI, and the porous and stable framework is expected to enlarge the external surface area of the conducting polymer and thus enhance its electrochemical performance. To date, Zr-MOFs have mostly been employed in such composites, and their applications are mostly for aqueous supercapacitors. Such MOF-conducting polymer composites with other tetravalent MOFs, such as Ti-MOFs and Ce-MOFs, are relatively rare in the literature; lots of opportunities should be there in utilizing such composites in electrocatalysis and redox-based electrochemical sensors.

Electrically insulating, stable, and porous MOFs can also serve as porous coatings on top of the active electrodes, to modulate the microenvironment for electrochemical processes, or to prevent the pore-confined nanoparticles near the MOF-electrode interface from agglomeration. With such a material design, the MOF itself is not necessary to be electrochemically active. The MOF coating may adjust the local concentrations of certain ionic species or modulate the ionic flux near the electrode surface. As a result, such a porous coating is capable of altering the reaction rates or selectivity for electrocatalysis or suppressing the dendrite formation on electrodes of aqueous batteries. Furthermore, the MOF coating may also adjust the energy barrier for forming certain surface-adsorbed intermediates on the neighboring surface of the electrocatalyst—a key to affecting the selectivity of electrocatalysis.^90^ But using such electrochemically “inactive” MOF coatings for electrochemical applications belongs to a relatively new concept, with limited numbers of studies mostly published over the past five years. The node connectivity, degree of defects, pore sizes, and functional groups on linkers of the MOF coating should play important roles, in either adjusting the mass transfer across the MOF, or facilitating the formation of desirable surface-adsorbed intermediates on the adjacent catalyst’s surface. However, these effects have not been systematically investigated yet. Besides, most studies in this subfield still employed Zr-MOFs, but it has been known that the type of metal-based clusters in the MOF coating can also play an important role,^92^ even though they are not electrochemically active. Ce-MOFs, Ti-MOF, Hf-MOF, or even Th-MOFs, should thus have opportunities to serve as electrochemically inactive porous coatings for selected electrocatalytic reactions or aqueous batteries.

As intrinsically insulators for electrons, these tetravalent MOFs are ideal candidates for the use in separators or gel/solid-state electrolytes—important components in batteries. Especially, since these MOFs are stable in acidic aqueous solutions, they are fully compatible with ZIBs and VFBs. The use of such MOFs as additives in separators of ZIBs and VFBs belongs to an emerging subfield, with most studies published over the past five years. To date, all published studies in this subfield used Zr-MOFs, and most of them still used UiO-66 and its analogs with various functional groups. For separators of aqueous batteries, the use of other structurally diverse Zr-MOFs, and the use of other tetravalent MOFs such as Ce-MOFs, Ti-MOFs and Hf-MOFs, have not been extensively investigated in the literature.

### 5.3 Adjusting Pore Sizes of MOFs to Modulate Mass Transfer

Mass transfer of ionic species in MOFs is important when MOFs are employed in most electrochemical applications. It should be considered for redox-active MOFs, since the redox conductivity is coupled with the mass transfer of counterions.^5,49^ For electrocatalysts confined within electrochemically inactive MOFs, the mass transfer of reactants through the composite is also crucial to achieve a high reaction rate. For MOF coatings on top of electrocatalysts, their mass transfer also matters, especially at a large overpotential. In addition, when MOFs are incorporated in nanocomposites as active materials for supercapacitors, the mass transfer of ions in the composite should directly affect the rate capability of the energy-storage device. Creating large pores in these tetravalent MOFs should thus be beneficial, if the fast mass transfer of ions in the MOF is desired. In addition to creating structurally derived MOF pores, which are usually below 5 nm in these tetravalent MOFs, utilizing soft-template-assisted approaches can create large and ordered mesopores with sizes of up to 40 nm in the MOF crystal.^127^ In our recent study, it was found that such large mesopores in a MOF deposited on electrodes can largely promote the mass transfer of reactants in the framework during electrocatalysis.^93^ We believe that such MOFs with hierarchical porosity and large mesopores should be beneficial for other applications such as supercapacitors and aqueous batteries as well, and the similar concept of generating large mesopores should be generalizable to Ce-MOFs, Ti-MOFs and Hf-MOFs, aiming for diverse electrochemical applications.

### 5.4 Stability of MOFs in Electrochemical Systems

Stability of the selected MOF in the targeted electrolyte, as well as that at the applied potential, always need to be considered. As discussed in the introduction, if the degradation of MOF occurs under certain electrochemical conditions, it is more appropriate to identify the MOF as a precursor or precatalyst, rather than the actual active material for a certain electrochemical application. The stability limitations of tetravalent MOFs in various aqueous solutions and buffers have been discussed in the introduction. In particular, it should be noted that, although Ce-MOFs possess electrochemical redox activity originating from the Ce^4+^/Ce^3+^ reaction of quite a minor proportion of cerium atoms in their clusters, the complete reduction of all cerium atoms in the MOF into Ce^3+^ should cause the degradation of the framework.^35^ Therefore, compared to Zr-MOFs and Hf-MOFs, extra care should be taken when exposing Ce-MOFs to reductants, since these MOFs are not that stable under such conditions. Utilizing tetravalent MOFs in strongly alkaline electrolytes usually results in framework degradation. For electrochemical applications requiring weakly alkaline electrolytes slightly beyond the limitation for Zr-MOFs, structurally similar Hf-MOFs and Th-MOFs with slightly better stability in alkaline solutions could be possible candidates. It should be noted that there are only a few studies reporting the use of Hf-MOFs in electrochemical applications, and the electrochemical applications of Th-MOFs have not been explored in any published work.

Several strategies may be used to further enhance the chemical stability of these tetravalent MOFs. For example, stability can be enhanced through judicious ligand selection; by choosing structurally rigid organic linkers, the hydrolytic robustness of tetravalent MOFs can be further boosted.^128^ In addition to linker engineering, introducing hydrophobic substituent groups, such as −F, −CF_3_ and alkyl groups, onto the framework, could be another effective strategy for reducing water accessibility and mitigating framework degradation.^129,130^ Moreover, integrating MOFs into composite architectures, such as MOF/carbon hybrids and polymer-capped MOF crystals, provides an additional pathway to mitigate structural degradation during long-term electrochemical cycling. These combined strategies represent a practical toolbox for improving the structural stability and thus electrochemical durability of tetravalent MOFs in harsh aqueous environments.

Finally, in addition to modulating the structural features of tetravalent MOFs and their composites and investigating their effects on electrochemical performance, *in situ* characterizations can be further employed for elucidating the metal–ligand coordination dynamics, defect evolution, and local structural rearrangements during the electrochemical operation.^131^ Furthermore, the integration of computational chemistry with machine learning offers powerful capabilities for analyzing complex reaction pathways and predicting structure–activity relationships.^132^ These advanced approaches provide promising opportunities and strategic directions for the future development of water-stable MOF-based electrochemical systems.
